# Chlorido­(2-{(2-hy­droxy­eth­yl)[tris­(hy­droxy­meth­yl)meth­yl]amino}­ethano­lato-κ^5^ *N*,*O*,*O*′,*O*′′,*O*′′′)copper(II)

**DOI:** 10.1107/S2414314624004395

**Published:** 2024-05-24

**Authors:** Monserrat Fortis-Valera, Rosa Elena Arroyo-Carmona, Aarón Pérez-Benítez, Sylvain Bernès

**Affiliations:** a Facultad de Ciencias Químicas, Benemérita Universidad Autónoma de Puebla, Ciudad Universitaria, 72570 Puebla, Pue., Mexico; b Instituto de Física, Benemérita Universidad Autónoma de Puebla, Av. San Claudio y 18 Sur, 72570 Puebla, Pue., Mexico; Vienna University of Technology, Austria

**Keywords:** crystal structure, coordination compound, bis-tris­, supra­molecular structure, very strong hydrogen bonds

## Abstract

The title complex, [Cu(C_8_H_18_NO_5_)Cl] or [Cu(H_4_bis-tris­)Cl], was obtained starting from the previously reported [Cu(H_5_bis-tris­)Cl]Cl compound. The deprotonation of the amino­polyol ligand H_5_bis-tris {[bis­(2-hy­droxy­eth­yl)amino]­tris­(hy­droxy­meth­yl)methane, C_8_H_19_NO_5_} promotes the formation of a very strong O—H⋯O inter­molecular hydrogen bond, characterized by an H⋯O separation of 1.553 (19) Å and an O—H⋯O angle of 178 (4)°. The remaining hy­droxy groups are also engaged in hydrogen bonds, forming *R*_2_^2^(8), *R*_4_^4^(16), *R*_4_^4^(20) and *R*_4_^4^(22) ring motifs, which stabilize the triperiodic supra­molecular network.

## Abstract 2

The crystal structure of the title complex features O—H⋯O hydrogen bonds, which form 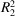 (8), 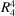 (16), 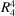 (20) and 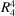 (22) ring motifs.

## Graphical abstract

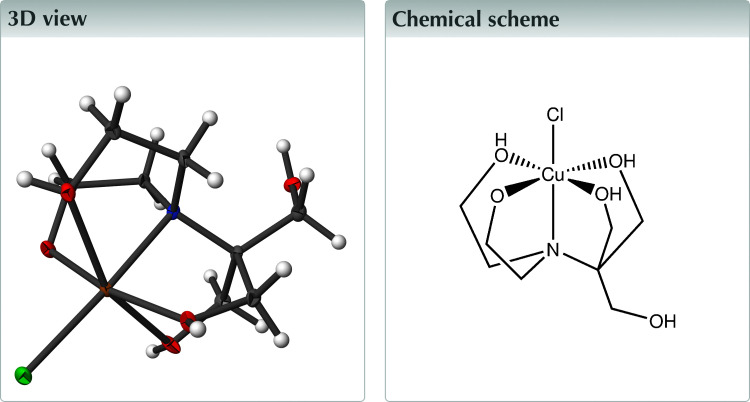

## Structure description

Amino­polyol [bis­(2-hy­droxy­eth­yl)amino]­tris­(hy­droxy­meth­yl)methane, generally abbrev­iated H_5_bis-tris­, is able to coordinate first-row late transition metals and lanthanides (Nicholson *et al.*, 2001 ▸). This mol­ecule behaves systematically as a chelating penta­dentate ligand, through the tertiary N atom and four of the five alcohol arms. The metal coordination sphere is then completed with an ancillary ligand, frequently an aqua or a chlorido ligand. Furthermore, depending on the reaction conditions, H_5_bis-tris can be deprotonated, affording chelating anions. While anions (H_5–*n*_bis-tris­)^*n*−^ with *n* = 2 to 4 have been determined by X-ray structure analysis in several compounds (*e.g*. Stamatatos *et al.*, 2009 ▸), it seems that to date the anionic ligand with *n* = 1, (H_4_bis-tris­)^−^, has been observed only once: Kirillova *et al.* (2017 ▸) reported a crystal structure comprising [Cu(H_5_bis-tris­)(inic)]^+^ and [Cu(H_4_bis-tris­)(inic)] entities, where inic stands for the isonicotinate anion. We now report the structure of the second complex where (H_4_bis-tris­)^−^ acts as a ligand, namely [Cu(H_4_bis-tris­)Cl], which was obtained serendipitously from [Cu(H_5_bis-tris­)Cl]^+^Cl (Inomata *et al.*, 2004 ▸).

**Figure 1:**
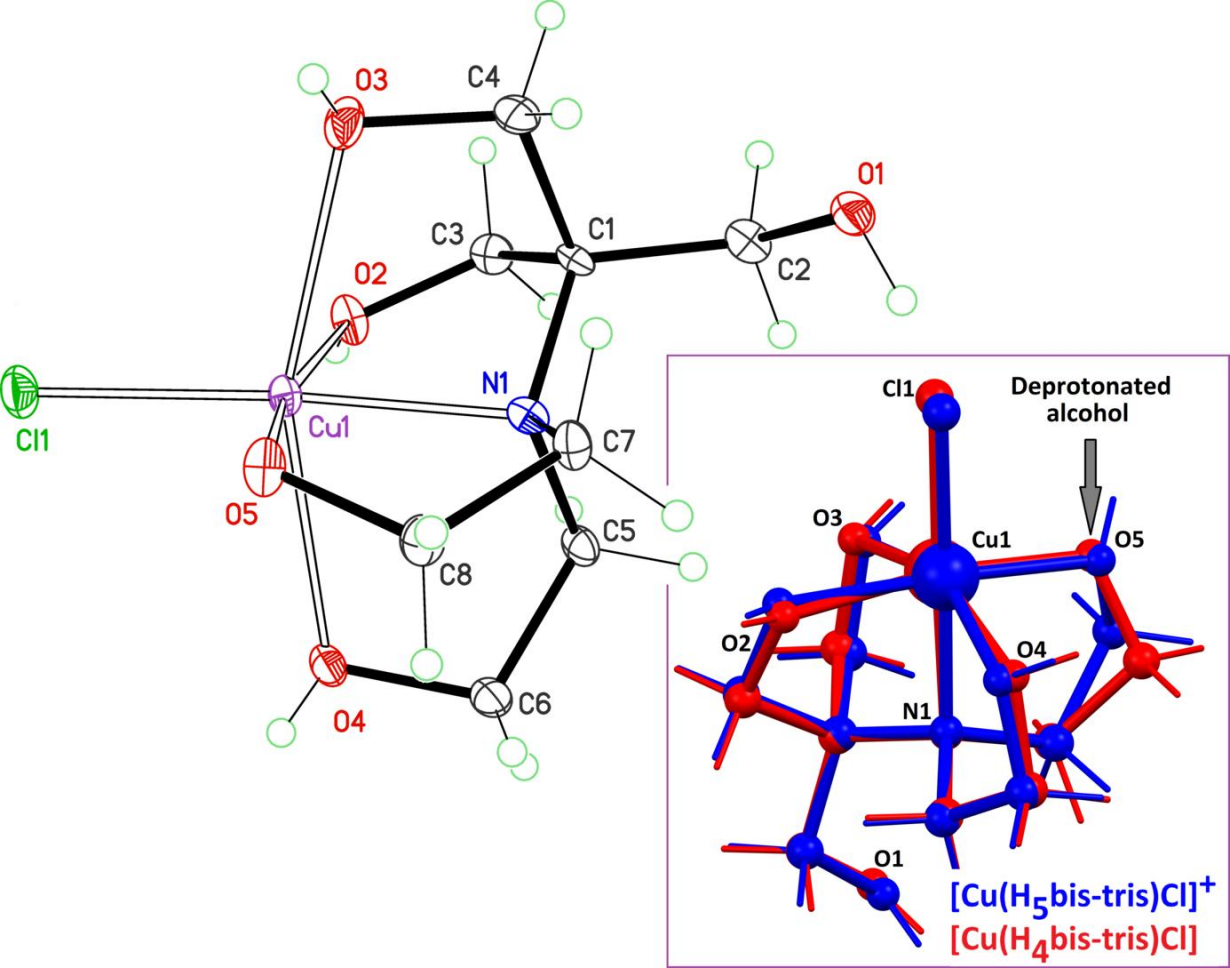
Mol­ecular structure of the title compound, with displacement ellipsoids for non-H atoms at the 60% probability level. The inset is an overlay calculated with Mercury (Macrae *et al.*, 2020 ▸), comparing the shape of [Cu(H_5_bis-tris­)Cl]^+^ (blue) and the title complex [Cu(H_4_bis-tris­)Cl] (red). The crystal structure of [Cu(H_5_bis-tris­)Cl]Cl has been published (Inomata *et al.*, 2004 ▸; CCDC refcode FIPRAY); however, the authors did not deposit a CIF file at that time. A CSD communication for this compound was thus used for the fit (FIPRAY01; Fortis-Valera *et al.*, 2018 ▸).

The new Cu^II^ mol­ecular complex displays the expected distorted octa­hedral shape (Fig. 1 ▸). Since all H atoms could be located from electron-difference maps, the deprotonated alcohol group was clearly identified as being O5. Moreover, the anion formula for (H_4_bis-tris­)^−^ is consistent with the charge balance in the complex. The tetra­gonal distortion resulting from the Jahn–Teller effect for Cu^II^ increases bond lengths Cu1—O3 and Cu1—O4 [2.361 (3) and 2.436 (2) Å] in comparison with bond lengths in the equatorial plane N1/O2/O5/Cl1 [1.943 (2) to 2.2812 (10) Å]. The shape of the neutral mol­ecule [Cu(H_4_bis-tris­)Cl] is actually close to that observed for the cation [Cu(H_5_bis-tris­)Cl]^+^: a mol­ecular overlay gives a root-mean-square (r.m.s.) deviation of 0.28 Å and a maximum deviation of 0.91 Å (Fig. 1 ▸, inset).

**Figure 2:**
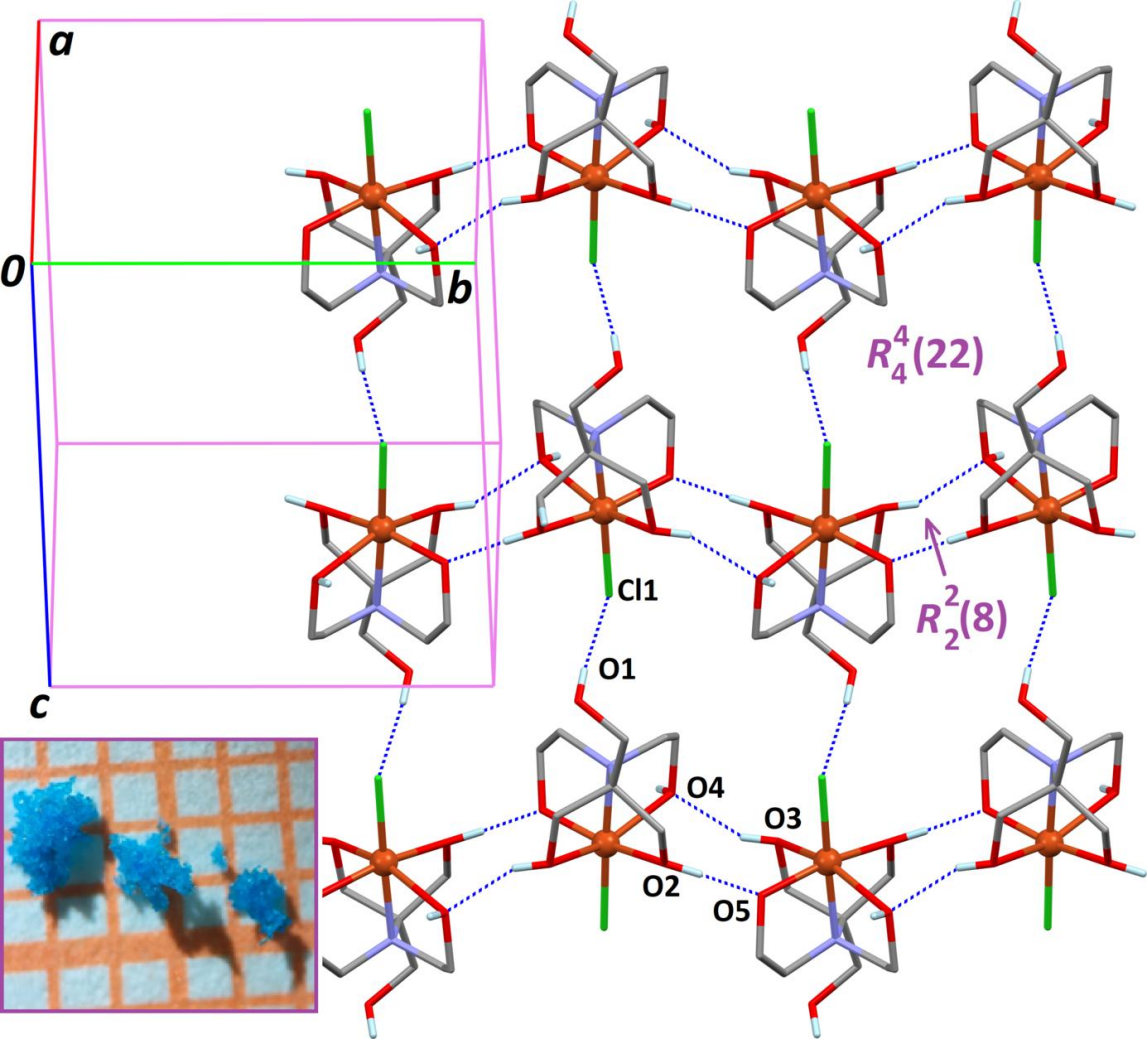
The supra­molecular framework based on inter­molecular O—H⋯(O, Cl) hydrogen bonds (dashed blue lines) corresponding to entries 1–3 in Table 1 ▸. The inset shows single crystals suitable for X-ray diffraction.

**Figure 3:**
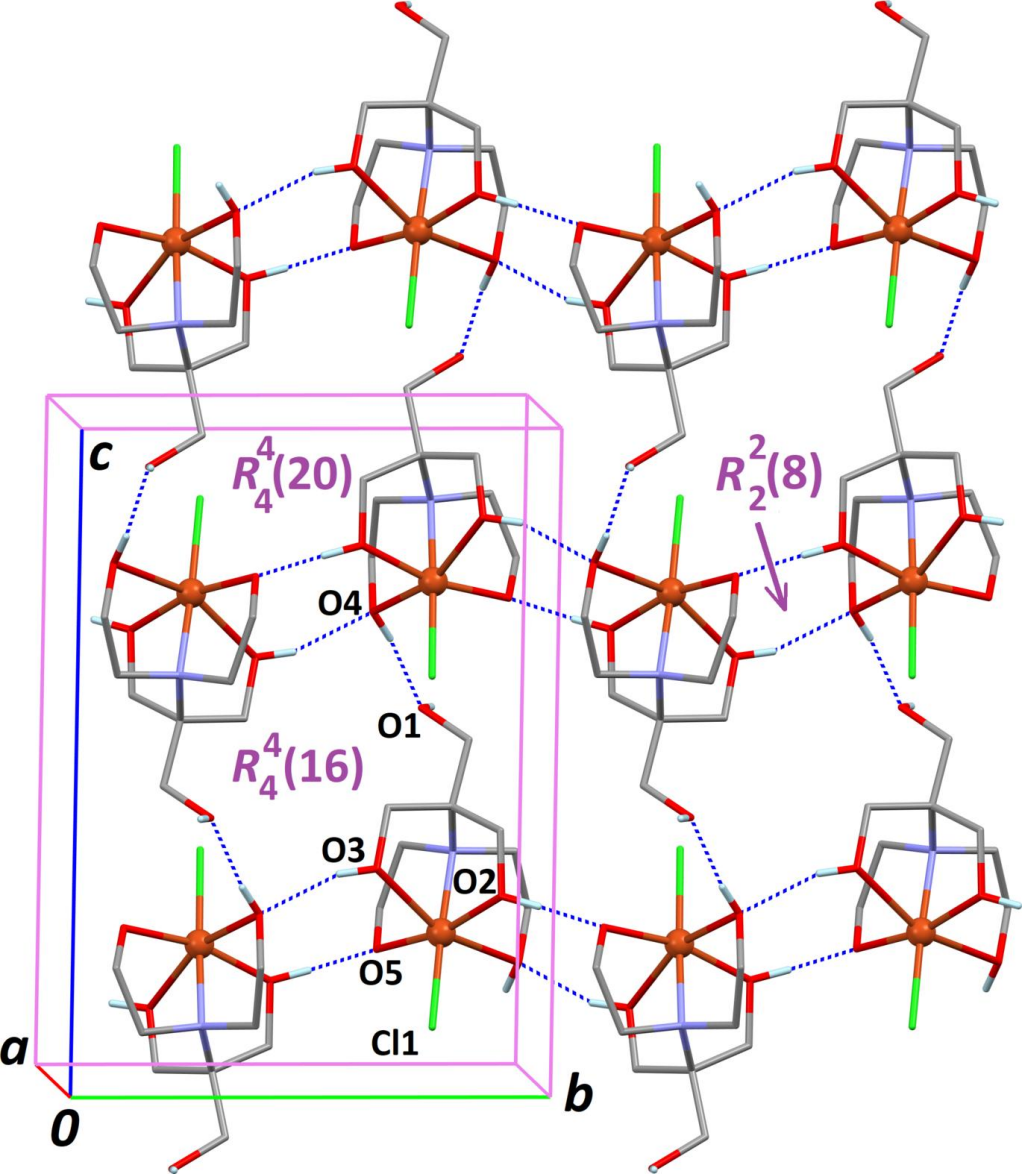
The supra­molecular framework based on inter­molecular O—H⋯O hydrogen bonds (dashed blue lines) corresponding to entries 2–4 in Table 1 ▸.

The space group and the network of hydrogen bonds are however modified upon deprotonation of H_5_bis-tris­. In the new complex, all hy­droxy groups are donors for hydrogen bonding, and the deprotonated hy­droxy group, O5, is an acceptor (Table 1 ▸). The latter is engaged in the strongest inter­action, O2—H2⋯O5, with a very short H2⋯O5 distance of 1.553 (19) Å and with an angle O2—H2⋯O5 = 178 (4)°. Indeed, only few shorter inter­molecular H⋯O separations can be retrieved from the Cambridge Structural Database (CSD v. 5.45, updated March 2024; Groom *et al.*, 2016 ▸) for CH_2_—CH_2_—O⋯H—O fragments (see, for example: Yilmaz *et al.*, 2002 ▸). Together with contact O3—H3⋯O4, 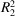 (8) ring motifs are formed in the crystal structure. Combined with another hydrogen bond involving the non-coordinating alcohol group, O1—H1⋯Cl1, a diperiodic framework is formed parallel to (101), based on 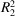 (8) and 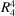 (22) supra­molecular motifs (Fig. 2 ▸). The last hydrogen bond, O4—H4⋯O1, expands the supra­molecular network through the formation of centrosymmetric 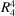 (16) and 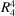 (20) rings (Fig. 3 ▸), affording a stable triperiodic crystal structure.

## Synthesis and crystallization

Single crystals of the title complex were unexpectedly obtained in an attempt to substitute the chlorido ligand in [Cu(H_5_bis-tris­)Cl]^+^ by a heterocyclic compound. Complex [Cu(H_5_bis-tris­)Cl]^+^Cl^−^ (1 mmol, 0.343 g) and fluconazole (1 mmol, 0.307 g) were dissolved in ethanol (70% *v*/*v* solution, 15 ml). The mixture was heated to 323 K under stirring for 20 min, and filtered to eliminate a blue precipitate. The resulting solution was evaporated over 3 days, affording a blue product. The crude product was recrystallized in methanol, giving sky-blue crystals used for the diffraction study (see Fig. 2 ▸, inset).

## Refinement

Crystal data, data collection and structure refinement details are summarized in Table 2 ▸.

## Supplementary Material

Crystal structure: contains datablock(s) I. DOI: 10.1107/S2414314624004395/wm4213sup1.cif

Structure factors: contains datablock(s) I. DOI: 10.1107/S2414314624004395/wm4213Isup2.hkl

CCDC reference: 2355145

Additional supporting information:  crystallographic information; 3D view; checkCIF report

## Figures and Tables

**Table 1:** Hydrogen-bond geometry (Å, °)

*D*—H⋯*A*	*D*—H	H⋯*A*	*D*⋯*A*	*D*—H⋯*A*
O1—H1⋯Cl1^i^	0.83 (2)	2.24 (2)	3.068 (2)	174 (4)
O2—H2⋯O5^ii^	0.86 (2)	1.55 (2)	2.408 (3)	178 (4)
O3—H3⋯O4^iii^	0.82 (2)	2.00 (2)	2.758 (3)	154 (4)
O4—H4⋯O1^iv^	0.82 (2)	1.85 (2)	2.663 (3)	171 (4)

Symmetry codes: (i) 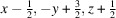 ; (ii) 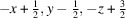 ; (iii) 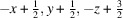 ; (iv) 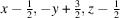 .

**Table 2:** Experimental details

Crystal data	
Chemical formula	[Cu(C_8_H_18_NO_5_)Cl]
*M*_r_	307.22
Crystal system, space group	Monoclinic, *P*2_1_/*n*
Temperature (K)	109
*a*, *b*, *c* (Å)	7.2605 (9), 10.4221 (14), 14.668 (2)
β (°)	94.366 (12)
*V* (Å^3^)	1106.7 (3)
*Z*	4
Radiation type	Mo *K*α
μ (mm^−1^)	2.22
Crystal size (mm)	0.17 × 0.06 × 0.04

Data collection	
Diffractometer	Xcalibur, Atlas, Gemini
Absorption correction	Analytical (*CrysAlis PRO*; Rigaku OD, 2022 ▸)
*T*_min_, *T*_max_	0.848, 0.917
No. of measured, independent and observed [*I* > 2σ(*I*)] reflections	5523, 2582, 2002
*R*_int_	0.046
(sin θ/λ)_max_ (Å^−1^)	0.694

Refinement	
*R*[*F* ^2^ > 2σ(*F* ^2^)], *wR*(*F* ^2^), *S*	0.048, 0.130, 1.05
No. of reflections	2582
No. of parameters	157
No. of restraints	4
H-atom treatment	H atoms treated by a mixture of independent and constrained refinement
Δρ_max_, Δρ_min_ (e Å^−3^)	1.18, −1.17

Computer programs: *CrysAlis PRO* (Rigaku OD, 2022 ▸), *SHELXT2018/2* (Sheldrick, 2015*a* ▸), *SHELXL2018/3* (Sheldrick, 2015*b* ▸), *XP* in *SHELXTL-Plus* (Sheldrick, 2008 ▸), *Mercury* (Macrae *et al.*, 2020 ▸) and *publCIF* (Westrip, 2010 ▸).

## full crystallographic data

### Crystal data

**Table:**

[Cu(C_8_H_18_NO_5_)Cl]	*D*_x_ = 1.844 Mg m^−^^3^
*M**_r_* = 307.22	Melting point: 435 K
Monoclinic, *P*2_1_/*n*	Mo *K*α radiation, λ = 0.71073 Å
*a* = 7.2605 (9) Å	Cell parameters from 1395 reflections
*b* = 10.4221 (14) Å	θ = 3.4–29.6°
*c* = 14.668 (2) Å	µ = 2.22 mm^−^^1^
β = 94.366 (12)°	*T* = 109 K
*V* = 1106.7 (3) Å^3^	Prism, blue
*Z* = 4	0.17 × 0.06 × 0.04 mm
*F*(000) = 636	

### Data collection

**Table:**

Xcalibur, Atlas, Gemini diffractometer	2582 independent reflections
Radiation source: Enhance (Mo) X-ray Source	2002 reflections with *I* > 2σ(*I*)
Graphite monochromator	*R*_int_ = 0.046
Detector resolution: 10.4685 pixels mm^-1^	θ_max_ = 29.6°, θ_min_ = 3.4°
ω scans	*h* = −9→10
Absorption correction: analytical (CrysAlisPro; Rigaku OD, 2022)	*k* = −14→13
*T*_min_ = 0.848, *T*_max_ = 0.917	*l* = −18→18
5523 measured reflections	

### Refinement

**Table:**

Refinement on *F*^2^	Primary atom site location: dual
Least-squares matrix: full	Secondary atom site location: difference Fourier map
*R*[*F*^2^ > 2σ(*F*^2^)] = 0.048	Hydrogen site location: mixed
*wR*(*F*^2^) = 0.130	H atoms treated by a mixture of independent and constrained refinement
*S* = 1.05	*w* = 1/[σ^2^(*F*_o_^2^) + (0.0646*P*)^2^] where *P* = (*F*_o_^2^ + 2*F*_c_^2^)/3
2582 reflections	(Δ/σ)_max_ = 0.001
157 parameters	Δρ_max_ = 1.18 e Å^−^^3^
4 restraints	Δρ_min_ = −1.17 e Å^−^^3^
0 constraints	

### Special details

**Table:**

Refinement. Methylene H atoms were refined using a riding model (C—H: 0.99 Å); hydroxy H atoms (H1, H2, H3, H4) were located from electron-difference maps and were refined freely, with the O—H bond lengths restrained to 0.85 (2) Å.

### Fractional atomic coordinates and isotropic or equivalent isotropic displacement parameters (Å^2^)

**Table:**

	*x*	*y*	*z*	*U*_iso_*/*U*_eq_	
Cu1	0.22842 (5)	0.75373 (4)	0.75931 (3)	0.00775 (16)	
Cl1	0.31899 (12)	0.76196 (8)	0.61387 (6)	0.0145 (2)	
O1	0.2269 (3)	0.8024 (2)	1.09572 (16)	0.0130 (5)	
H1	0.116 (3)	0.790 (4)	1.103 (3)	0.019*	
O2	0.3908 (3)	0.6168 (2)	0.80600 (16)	0.0096 (5)	
H2	0.407 (5)	0.539 (2)	0.790 (3)	0.014*	
O3	0.4592 (3)	0.8730 (2)	0.84389 (17)	0.0143 (5)	
H3	0.462 (5)	0.9507 (19)	0.836 (3)	0.022*	
O4	−0.0338 (3)	0.6104 (2)	0.72784 (16)	0.0104 (5)	
H4	−0.113 (4)	0.641 (4)	0.692 (2)	0.016*	
O5	0.0620 (3)	0.8995 (2)	0.74310 (16)	0.0111 (5)	
N1	0.1319 (4)	0.7390 (2)	0.8869 (2)	0.0075 (6)	
C1	0.3128 (5)	0.7294 (3)	0.9443 (2)	0.0080 (7)	
C2	0.2964 (5)	0.6976 (3)	1.0446 (2)	0.0121 (7)	
H2A	0.213089	0.622927	1.048817	0.015*	
H2B	0.419525	0.672641	1.072706	0.015*	
C3	0.4235 (4)	0.6194 (3)	0.9021 (2)	0.0109 (7)	
H3A	0.557130	0.631837	0.918562	0.013*	
H3B	0.386836	0.536144	0.927768	0.013*	
C4	0.4244 (4)	0.8542 (3)	0.9372 (2)	0.0104 (7)	
H4A	0.353726	0.927666	0.959330	0.012*	
H4B	0.542532	0.847779	0.975361	0.012*	
C5	0.0232 (4)	0.6181 (3)	0.8917 (2)	0.0096 (7)	
H5A	−0.039617	0.617991	0.949336	0.012*	
H5B	0.109414	0.544400	0.893907	0.012*	
C6	−0.1206 (4)	0.5993 (3)	0.8123 (2)	0.0118 (7)	
H6A	−0.178021	0.513531	0.816363	0.014*	
H6B	−0.218735	0.664857	0.814672	0.014*	
C7	0.0172 (4)	0.8544 (3)	0.9019 (2)	0.0094 (7)	
H7A	0.095096	0.922177	0.932473	0.011*	
H7B	−0.081487	0.832157	0.942142	0.011*	
C8	−0.0692 (4)	0.9044 (3)	0.8101 (2)	0.0114 (7)	
H8A	−0.177933	0.851406	0.789966	0.014*	
H8B	−0.111298	0.993927	0.817219	0.014*	

### Atomic displacement parameters (Å^2^)

**Table:**

	*U*^11^	*U*^22^	*U*^33^	*U*^12^	*U*^13^	*U*^23^
Cu1	0.0099 (3)	0.0069 (2)	0.0065 (2)	0.00093 (15)	0.00096 (17)	0.00036 (15)
Cl1	0.0135 (4)	0.0219 (5)	0.0084 (4)	0.0024 (3)	0.0025 (3)	0.0011 (3)
O1	0.0108 (11)	0.0173 (13)	0.0108 (13)	−0.0005 (11)	0.0007 (9)	−0.0040 (11)
O2	0.0163 (11)	0.0046 (11)	0.0080 (12)	0.0047 (10)	0.0018 (9)	−0.0025 (10)
O3	0.0218 (12)	0.0108 (11)	0.0110 (13)	−0.0061 (11)	0.0049 (10)	0.0006 (11)
O4	0.0128 (11)	0.0111 (12)	0.0073 (12)	−0.0009 (10)	−0.0007 (9)	0.0000 (10)
O5	0.0154 (11)	0.0085 (11)	0.0100 (12)	0.0031 (10)	0.0037 (9)	0.0011 (10)
N1	0.0095 (13)	0.0039 (13)	0.0088 (14)	0.0006 (10)	−0.0011 (11)	−0.0011 (11)
C1	0.0085 (15)	0.0082 (15)	0.0070 (16)	−0.0009 (13)	−0.0022 (12)	−0.0015 (13)
C2	0.0128 (16)	0.0119 (16)	0.0114 (18)	0.0007 (14)	−0.0012 (13)	−0.0009 (15)
C3	0.0114 (15)	0.0090 (16)	0.0121 (18)	0.0007 (14)	−0.0011 (12)	0.0020 (14)
C4	0.0120 (15)	0.0078 (16)	0.0112 (18)	−0.0019 (14)	0.0001 (12)	−0.0028 (13)
C5	0.0119 (15)	0.0090 (15)	0.0075 (16)	−0.0004 (14)	−0.0013 (12)	0.0018 (14)
C6	0.0137 (15)	0.0113 (16)	0.0101 (17)	−0.0055 (14)	−0.0015 (12)	0.0021 (14)
C7	0.0102 (15)	0.0105 (16)	0.0076 (17)	0.0038 (13)	0.0010 (12)	0.0011 (13)
C8	0.0124 (15)	0.0115 (16)	0.0102 (17)	0.0004 (14)	0.0012 (12)	0.0014 (14)

### Geometric parameters (Å, º)

**Table:**

Cu1—O2	1.943 (2)	C1—C4	1.540 (4)
Cu1—O5	1.944 (2)	C1—C3	1.556 (5)
Cu1—N1	2.054 (3)	C2—H2A	0.9900
Cu1—Cl1	2.2812 (10)	C2—H2B	0.9900
Cu1—O3	2.361 (3)	C3—H3A	0.9900
Cu1—O4	2.436 (2)	C3—H3B	0.9900
O1—C2	1.438 (4)	C4—H4A	0.9900
O1—H1	0.833 (19)	C4—H4B	0.9900
O2—C3	1.412 (4)	C5—C6	1.516 (4)
O2—H2	0.856 (19)	C5—H5A	0.9900
O3—C4	1.425 (4)	C5—H5B	0.9900
O3—H3	0.818 (19)	C6—H6A	0.9900
O4—C6	1.437 (4)	C6—H6B	0.9900
O4—H4	0.819 (18)	C7—C8	1.534 (5)
O5—C8	1.420 (4)	C7—H7A	0.9900
N1—C7	1.489 (4)	C7—H7B	0.9900
N1—C5	1.491 (4)	C8—H8A	0.9900
N1—C1	1.509 (4)	C8—H8B	0.9900
C1—C2	1.522 (5)		
			
O2—Cu1—O5	166.42 (10)	C1—C2—H2A	108.9
O2—Cu1—N1	82.17 (10)	O1—C2—H2B	108.9
O5—Cu1—N1	85.29 (10)	C1—C2—H2B	108.9
O2—Cu1—Cl1	98.45 (7)	H2A—C2—H2B	107.7
O5—Cu1—Cl1	94.41 (7)	O2—C3—C1	111.0 (3)
N1—Cu1—Cl1	176.13 (8)	O2—C3—H3A	109.4
O2—Cu1—O3	79.29 (9)	C1—C3—H3A	109.4
O5—Cu1—O3	93.57 (9)	O2—C3—H3B	109.4
N1—Cu1—O3	80.70 (10)	C1—C3—H3B	109.4
Cl1—Cu1—O3	103.18 (7)	H3A—C3—H3B	108.0
O2—Cu1—O4	93.48 (9)	O3—C4—C1	108.3 (3)
O5—Cu1—O4	89.25 (9)	O3—C4—H4A	110.0
N1—Cu1—O4	79.10 (10)	C1—C4—H4A	110.0
Cl1—Cu1—O4	97.04 (6)	O3—C4—H4B	110.0
O3—Cu1—O4	159.29 (9)	C1—C4—H4B	110.0
C2—O1—H1	110 (3)	H4A—C4—H4B	108.4
C3—O2—Cu1	112.91 (19)	N1—C5—C6	114.1 (3)
C3—O2—H2	106 (3)	N1—C5—H5A	108.7
Cu1—O2—H2	134 (3)	C6—C5—H5A	108.7
C4—O3—Cu1	105.16 (18)	N1—C5—H5B	108.7
C4—O3—H3	106 (3)	C6—C5—H5B	108.7
Cu1—O3—H3	118 (3)	H5A—C5—H5B	107.6
C6—O4—Cu1	105.88 (18)	O4—C6—C5	109.3 (3)
C6—O4—H4	105 (3)	O4—C6—H6A	109.8
Cu1—O4—H4	113 (3)	C5—C6—H6A	109.8
C8—O5—Cu1	112.74 (19)	O4—C6—H6B	109.8
C7—N1—C5	111.8 (3)	C5—C6—H6B	109.8
C7—N1—C1	116.3 (3)	H6A—C6—H6B	108.3
C5—N1—C1	111.1 (2)	N1—C7—C8	109.9 (3)
C7—N1—Cu1	107.9 (2)	N1—C7—H7A	109.7
C5—N1—Cu1	109.0 (2)	C8—C7—H7A	109.7
C1—N1—Cu1	99.8 (2)	N1—C7—H7B	109.7
N1—C1—C2	115.2 (3)	C8—C7—H7B	109.7
N1—C1—C4	110.3 (3)	H7A—C7—H7B	108.2
C2—C1—C4	109.3 (3)	O5—C8—C7	110.1 (3)
N1—C1—C3	106.3 (3)	O5—C8—H8A	109.6
C2—C1—C3	107.7 (3)	C7—C8—H8A	109.6
C4—C1—C3	107.8 (3)	O5—C8—H8B	109.6
O1—C2—C1	113.2 (3)	C7—C8—H8B	109.6
O1—C2—H2A	108.9	H8A—C8—H8B	108.1
			
C7—N1—C1—C2	72.7 (4)	C4—C1—C3—O2	−82.8 (3)
C5—N1—C1—C2	−56.7 (4)	Cu1—O3—C4—C1	22.4 (3)
Cu1—N1—C1—C2	−171.5 (2)	N1—C1—C4—O3	−60.1 (3)
C7—N1—C1—C4	−51.5 (4)	C2—C1—C4—O3	172.3 (3)
C5—N1—C1—C4	179.1 (3)	C3—C1—C4—O3	55.5 (3)
Cu1—N1—C1—C4	64.2 (3)	C7—N1—C5—C6	69.7 (4)
C7—N1—C1—C3	−168.1 (3)	C1—N1—C5—C6	−158.5 (3)
C5—N1—C1—C3	62.5 (3)	Cu1—N1—C5—C6	−49.5 (3)
Cu1—N1—C1—C3	−52.3 (3)	Cu1—O4—C6—C5	−28.5 (3)
N1—C1—C2—O1	−72.4 (3)	N1—C5—C6—O4	53.5 (4)
C4—C1—C2—O1	52.4 (4)	C5—N1—C7—C8	−91.3 (3)
C3—C1—C2—O1	169.2 (2)	C1—N1—C7—C8	139.7 (3)
Cu1—O2—C3—C1	2.2 (3)	Cu1—N1—C7—C8	28.6 (3)
N1—C1—C3—O2	35.5 (3)	Cu1—O5—C8—C7	34.4 (3)
C2—C1—C3—O2	159.4 (3)	N1—C7—C8—O5	−41.6 (4)

### Hydrogen-bond geometry (Å, º)

**Table:**

*D*—H···*A*	*D*—H	H···*A*	*D*···*A*	*D*—H···*A*
O1—H1···Cl1^i^	0.83 (2)	2.24 (2)	3.068 (2)	174 (4)
O2—H2···O5^ii^	0.86 (2)	1.55 (2)	2.408 (3)	178 (4)
O3—H3···O4^iii^	0.82 (2)	2.00 (2)	2.758 (3)	154 (4)
O4—H4···O1^iv^	0.82 (2)	1.85 (2)	2.663 (3)	171 (4)
C4—H4*B*···Cl1^v^	0.99	2.97	3.906 (3)	157
C5—H5*A*···Cl1^i^	0.99	2.97	3.887 (4)	155
C7—H7*B*···Cl1^i^	0.99	2.85	3.727 (3)	148
C8—H8*B*···O1^vi^	0.99	2.65	3.577 (4)	157

Symmetry codes: (i) *x*−1/2, −*y*+3/2, *z*+1/2; (ii) −*x*+1/2, *y*−1/2, −*z*+3/2; (iii) −*x*+1/2, *y*+1/2, −*z*+3/2; (iv) *x*−1/2, −*y*+3/2, *z*−1/2; (v) *x*+1/2, −*y*+3/2, *z*+1/2; (vi) −*x*, −*y*+2, −*z*+2.

## References

[bb1] Fortis-Valera, M., Bernès, S., Arroyo-Carmona, R. E. & Pérez-Benítez, A. (2018). CSD Communication (refcode FIPRAY01). CCDC, Cambridge, England

[bb2] Groom, C. R., Bruno, I. J., Lightfoot, M. P. & Ward, S. C. (2016). *Acta Cryst.* B**72**, 171–179. 10.1107/S2052520616003954 PMC4822653 27048719

[bb3] Inomata, Y., Gochou, Y., Nogami, M., Howell, F. S. & Takeuchi, T. (2004). *J. Mol. Struct.* **702**, 61–70.

[bb4] Kirillova, M. V., Santos, C. I. M., André, V., Fernandes, T. A., Dias, S. S. P. & Kirillov, A. M. (2017). *Inorg. Chem. Front.* **4**, 968–977.

[bb5] Macrae, C. F., Sovago, I., Cottrell, S. J., Galek, P. T. A., McCabe, P., Pidcock, E., Platings, M., Shields, G. P., Stevens, J. S., Towler, M. & Wood, P. A. (2020). *J. Appl. Cryst.* **53**, 226–235. 10.1107/S1600576719014092 PMC6998782 32047413

[bb6] Nicholson, K. N., Twamley, B. & Wood, S. (2001). *Acta Cryst.* E**57**, o1133–o1135.

[bb7] Rigaku OD (2022). *CrysAlis PRO*. Rigaku Oxford Diffraction Ltd, Yarnton, England.

[bb8] Sheldrick, G. M. (2008). *Acta Cryst.* A**64**, 112–122. 10.1107/S0108767307043930 18156677

[bb9] Sheldrick, G. M. (2015*a*). *Acta Cryst.* A**71**, 3–8.

[bb10] Sheldrick, G. M. (2015*b*). *Acta Cryst.* C**71**, 3–8.

[bb11] Stamatatos, T. C., Abboud, K. A. & Christou, G. (2009). *Dalton Trans.* pp. 41–50. 10.1039/b810701g 19081970

[bb12] Westrip, S. P. (2010). *J. Appl. Cryst.* **43**, 920–925.

[bb13] Yilmaz, V. T., Andac, O., Karadag, A. & Harrison, W. T. A. (2002). *J. Mol. Struct.* **641**, 119–124.

